# Supplementary figures and images for: Computational Modeling of C-Terminal Tails to Predict the Calcium-Dependent Secretion of Endoplasmic Reticulum Resident Proteins
Source: Front Chem. 2021 Jun 29;9:689608. doi: 10.3389/fchem.2021.689608 (PMC8276033; doi:10.3389/fchem.2021.689608)

Supplementary Figure S2

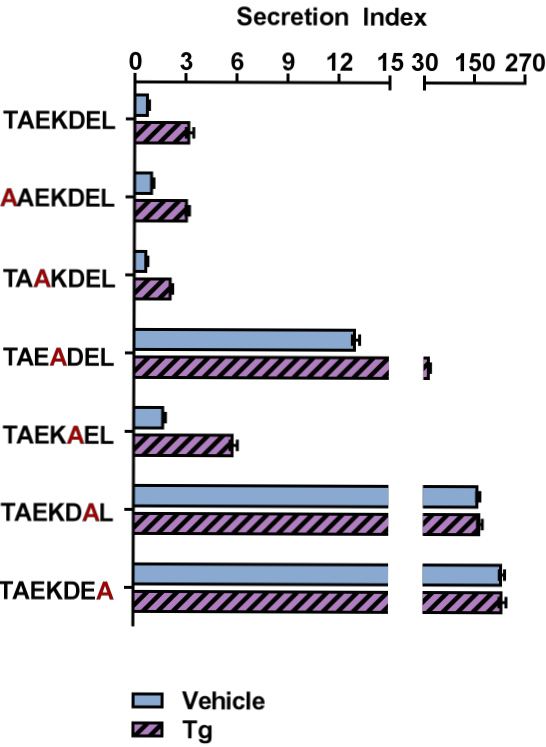

Supplement: Supplementary file 1 [file DataSheet2.PDF]

Supplementary Figure S4

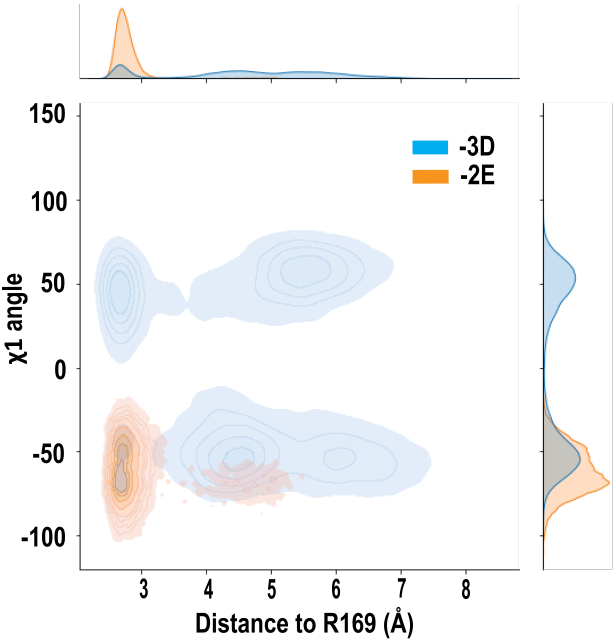

Supplement: Supplementary file 2 [file DataSheet4.PDF]

### Supplementary Figure S3

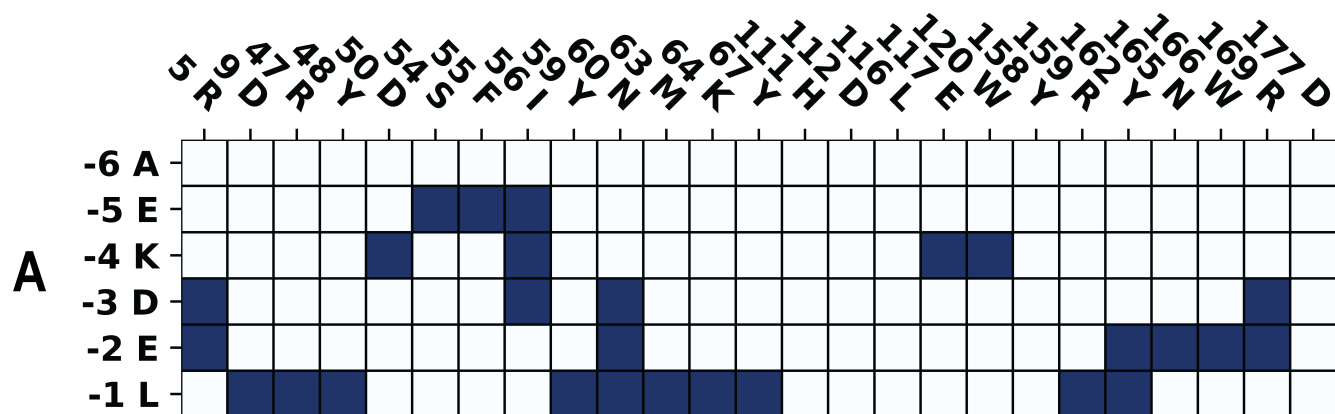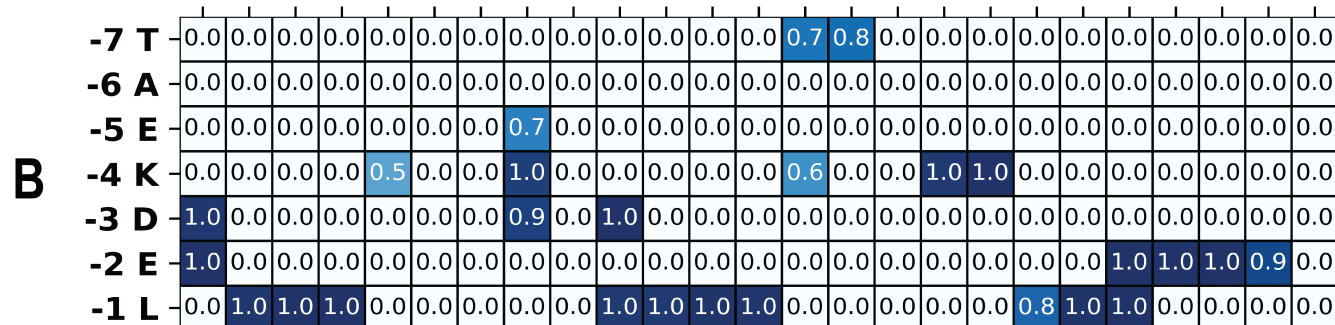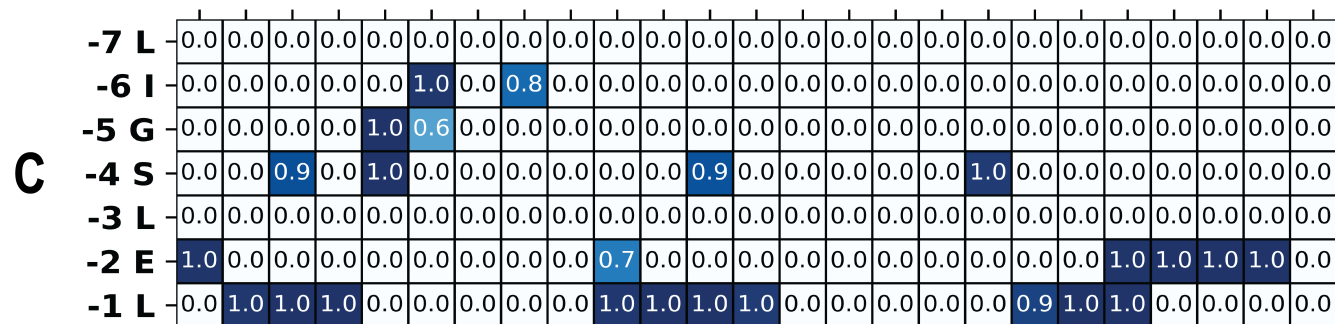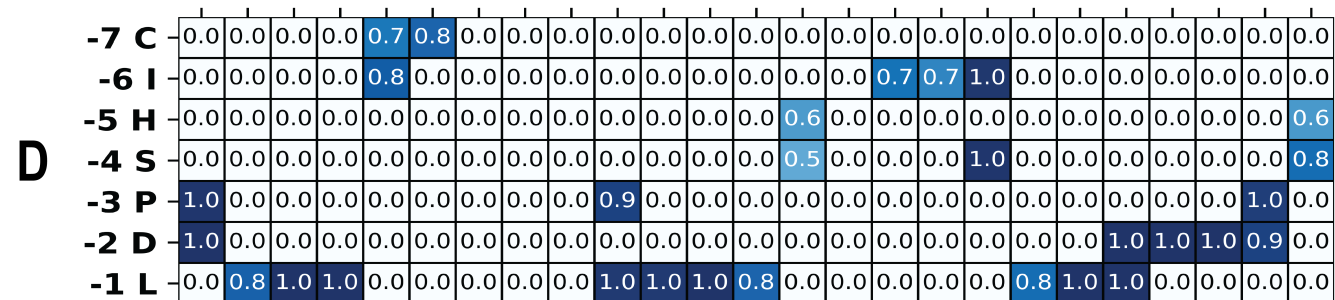

Supplement: Supplementary file 5 [file DataSheet3.PDF]

Supplementary Figure S1

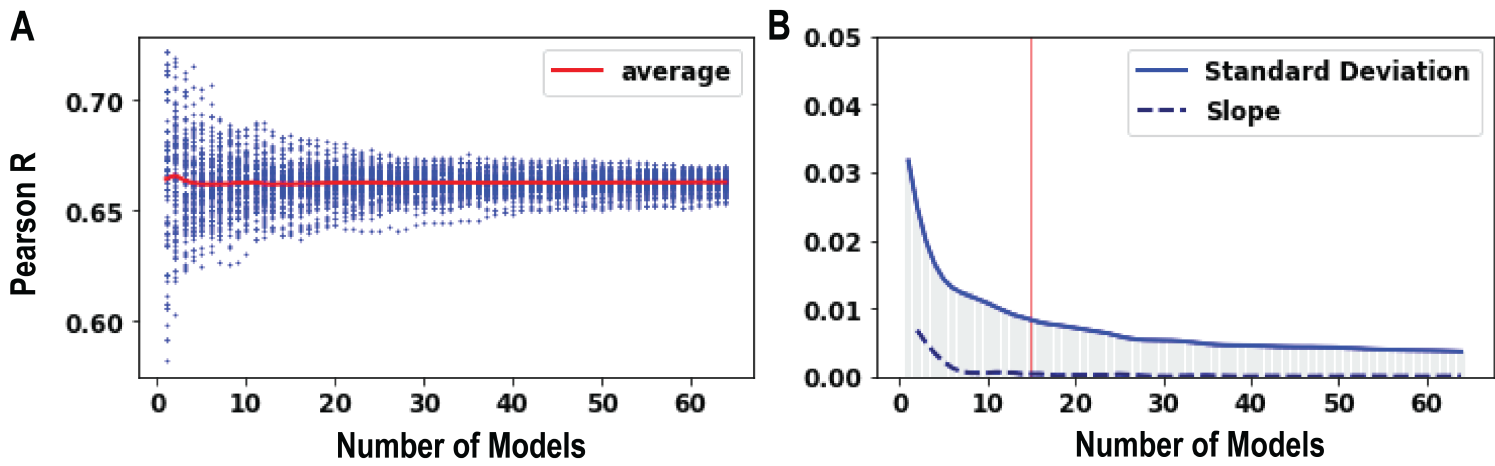

Supplement: Supplementary file 7 [file DataSheet1.PDF]
