# Supplementary material for: Computational Modeling of C-Terminal Tails to Predict the Calcium-Dependent Secretion of Endoplasmic Reticulum Resident Proteins
Source: Front Chem. 2021 Jun 29;9:689608. doi: 10.3389/fchem.2021.689608 (PMC8276033; doi:10.3389/fchem.2021.689608)
Supplement: Supplementary file 4 [file Table1.DOCX]

**Supplementary Table S1: ERS proteins are increased in the extracellular fluid in disease states.**

| Gene | Protein^1^ | Model | C-terminus | Measurement | Ref |
| --- | --- | --- | --- | --- | --- |
| Diabetes | | | | | |
| MANF | Mesencephalic astrocyte derived neurotrophic factor | Children with recently diagnosed Type 1 diabetes | ASARTDL | ELISA of serum | (Galli et al., 2016) |
| MANF | Mesencephalic astrocyte derived neurotrophic factor | Patients with Type 2 diabetes or newly diagnosed prediabetes | ASARTDL | ELISA of serum | (Wu et al., 2017) |
| SERPINH1/  Hsp47 | Serpin H1 | Diabetic patients with and without foot ulcers | DKMRDEL | ELISA of plasma | (Zubair and Ahmad, 2015) |
| BiP/Grp78 | Binding-immunoglobulin protein | Patients with Type 2 diabetes | TAEKDEL | ELISA of serum | (Girona et al., 2019) |
| Myeloproliferative (MF) Disorders | | | | | |
| CALR | Calreticulin | Patients with MF in chronic phase | GQAKDEL | ELISA of plasma | (Sollazzo et al., 2016) |
| Rheumatoid Arthritis (RA) | | | | | |
| BiP/Grp78 | Binding-immunoglobulin protein | RA patients | TAEKDEL | 2-DE, MS, Western blot of whole saliva | (Giusti et al., 2010) |
| CALR | Calreticulin | RA patients | GQAKDEL | ELISA of plasma and synovial fluid | (Tarr et al., 2010) |
| TXNDC5 | Thioredoxin domain-containing protein 5 | RA patients | SQAKDEL | ELISA of serum | (Chang et al., 2011) |
| Smoking, Asthma, and Chronic Obstructive Pulmonary Disease (COPD) | | | | | |
| BiP/Grp78 | Binding-immunoglobulin protein | Chronic smokers | TAEKDEL | Western blot of bronchoalveolar lavage fluid | (Aksoy et al., 2017) |
| BiP/Grp78 | Binding-immunoglobulin protein | Human airway epithelial cells treated with cigarette smoke extract (CSE) | TAEKDEL | Western blot of cell culture media | (Aksoy et al., 2017) |
| BiP/Grp78 | Binding-immunoglobulin protein | Ex-smokers with or without airflow obstruction | TAEKDEL | GeLC-MS/MS and Western blot of plasma | (Merali et al., 2014) |
| PDIA3 | Protein disulfide isomerase A3 | Asthma patients who are current smokers, ex-smokers, or non-smokers | KKAQEDL | Proteomic analysis of sputum | (Takahashi et al., 2018) |
| CALR | Calreticulin | Asthma patients who are current smokers, ex-smokers, or non-smokers | GQAKDEL | Proteomic analysis of sputum | (Takahashi et al., 2018) |
| Erp29 | Endoplasmic reticulum resident protein 29 | Asthma patients who are current smokers, ex-smokers, or non-smokers | GAEKEEL | Proteomic analysis of sputum | (Takahashi et al., 2018) |
| AGR2 | Anterior gradient protein 2 | Asthma patients who are current smokers, ex-smokers, or non-smokers | KLLKTEL | Proteomic analysis of sputum | (Takahashi et al., 2018) |
| LRPAP1 | Alpha-2-macroglobulin receptor-associated protein | Asthma patients who are current smokers, ex-smokers, or non-smokers | RARHNEL | Proteomic analysis of sputum | (Takahashi et al., 2018) |
| Ischemia |  |  | | | |
| PDIA2 | Protein disulfide isomerase A2 | Oxygen glucose deprivation in SH-SY5Y cells | MGSKEEL | Immunoprecipitation of cell culture media | (Trychta et al., 2018) |
| MANF | Mesencephalic astrocyte derived neurotrophic factor | Oxygen glucose deprivation in SH-SY5Y cells | ASARTDL | Homogeneous time resolved fluorescence assay (HTRF) of cell culture media | (Trychta et al., 2018) |
| MYDGF | Myeloid-derived growth factor | Myocardial infarction surgery in mice | KAARSEL | LC-MS of plasma | (Korf-Klingebiel et al., 2015) |
| MYDGF | Myeloid-derived growth factor | Patients with acute myocardial infarction | KASRTEL | LC-MS of plasma | (Korf-Klingebiel et al., 2015) |
| MYDGF | Myeloid-derived growth factor | Patients with myocardial infarction | KASRTEL | LC/MRM-MS of plasma | (Polten et al., 2018) |
| SERPINI1 | Neuroserpin | Patients with acute ischemic stroke | GHDFEEL | ELISA of serum | (Rodríguez-González et al., 2011) |
| Metabolic Syndrome | | | | | |
| PDIA4 | Protein disulfide isomerase A4 | Enrolled adults separated by rating on National Cholesterol Education Program Adult Treatment Panel III criteria for Asia Pacific | SRTKEEL | ELISA of serum | (Chien et al., 2017) |
| BiP/Grp78 | Binding-immunoglobulin protein | Patients with metabolic syndrome | TAEKDEL | ELISA of serum | (Girona et al., 2019) |
| Cancer | | | | | |
| BiP/Grp78 | Binding-immunoglobulin protein | Conditioned media from colon cancer cell lines | TAEKDEL | Western blot of concentrated cell culture media | (Fu et al., 2014) |
| AGR2 | Anterior gradient protein 2 | Patients with metastatic prostate cancer | KLLKTEL | ELISA of plasma | (Kani et al., 2013) |
| AGR2 | Anterior gradient protein 2 | Women at the time of ovarian cancer diagnosis | KLLKTEL | ELISA of plasma | (Edgell et al., 2010) |
| AGR2 | Anterior gradient protein 2 | Pancreatic cancer patients | KLLKTEL | ELISA of plasma | (Makawita et al., 2011) |
| CALR | Calreticulin | Lung cancer patients | GQAKDEL | Chemiluminescent enzyme immunoassay (CLEIA) of serum | (Liu et al., 2012) |
| C4BPA | C4b-binding protein alpha chain | Patients with pancreatic ductal adenocarcinoma | STLDKEL | ELISA of serum | (Sogawa et al., 2016) |
| Pneumonia | | | | | |
| SERPINH1/  Hsp47 | Serpin H1 | Patients with pneumonia | DKMRDEL | ELISA of serum | (Kakugawa et al., 2014) |
| PDIA3 | Protein disulfide isomerase A3 | Patients with acute lung injury with or without ventilator-associated pneumonia | KKAQEDL | HPLC-MS/MS of bronchoalveolar lavage fluid | (Nguyen et al., 2013) |
| Sclerosis | | | | | |
| SERPINH1/  Hsp47 | Serpin H1 | Chinese patients with systemic sclerosis | DKMRDEL | ELISA of plasma | (Chu et al., 2015) |
| Kidney Disease | | | | | |
| MANF | Mesencephalic astrocyte derived neurotrophic factor | Mice with nephrotic defects | ASARTDL | Western blot of urine | (Kim et al., 2016) |
| Takayasu arteritis (TA) | | | | | |
| C4BPA | C4b-binding protein alpha chain | Active-phase TA patients | STLDKEL | ELISA of plasma | (Ma et al., 2010) |
| Lupus | | | | | |
| CALR | Calreticulin | Patients with systemic lupus erythematosus, | GQAKDEL | ELISA of serum | (Wang et al., 2017) |
| Acute Lung Injury | | | | | |
| HSP90B1 | Endoplasmin/  Grp94 | Patients with acute lung injury | TAEKDEL | HPLC-MS/MS of BALF | (Nguyen et al., 2013) |

ELISA, enzyme linked immunosorbent assay; 2-DE, two-dimensional gel electrophoresis; MS, mass spectrometry; GeLC-MS/MS, gel electrophoresis followed by liquid chromatography-tandem mass spectrometry; LC-MS, liquid chromatrography-mass spectrometry; LC/MRM-MS, liquid chromatography/multiple reaction monitoring-mass spectrometry; HPLC-MS/MS, high-performance liquid chromatography tandem mass spectrometry

^1^ Protein name reflects Uniprot recommended name.

**References**

AKSOY, M. O., KIM, V., CORNWELL, W. D., ROGERS, T. J., KOSMIDER, B., BAHMED, K., BARRERO, C., MERALI, S., SHETTY, N. & KELSEN, S. G. 2017. Secretion of the endoplasmic reticulum stress protein, GRP78, into the BALF is increased in cigarette smokers. *Respiratory Research,* 18**,** 78.

CHANG, X., ZHAO, Y., YAN, X., PAN, J., FANG, K. & WANG, L. 2011. Investigating a pathogenic role for TXNDC5 in rheumatoid arthritis. *Arthritis research & therapy,* 13**,** R124-R124.

CHIEN, C.-Y., HUNG, Y.-J., SHIEH, Y.-S., HSIEH, C.-H., LU, C.-H., LIN, F.-H., SU, S.-C. & LEE, C.-H. 2017. A novel potential biomarker for metabolic syndrome in Chinese adults: Circulating protein disulfide isomerase family A, member 4. *PloS one,* 12**,** e0179963-e0179963.

CHU, H., WU, T., WU, W., TU, W., JIANG, S., CHEN, S., MA, Y., LIU, Q., ZHOU, X., JIN, L. & WANG, J. 2015. Involvement of collagen-binding heat shock protein 47 in scleroderma-associated fibrosis. *Protein & cell,* 6**,** 589-598.

EDGELL, T. A., BARRACLOUGH, D. L., RAJIC, A., DHULIA, J., LEWIS, K. J., ARMES, J. E., BARRACLOUGH, R., RUDLAND, P. S., RICE, G. E. & AUTELITANO, D. J. 2010. Increased plasma concentrations of anterior gradient 2 protein are positively associated with ovarian cancer. *Clin Sci (Lond),* 118**,** 717-25.

FU, R., YANG, P., WU, H. L., LI, Z. W. & LI, Z. Y. 2014. GRP78 secreted by colon cancer cells facilitates cell proliferation via PI3K/Akt signaling. *Asian Pac J Cancer Prev,* 15**,** 7245-9.

GALLI, E., HARKONEN, T., SAINIO, M. T., USTAV, M., TOOTS, U., URTTI, A., YLIPERTTULA, M., LINDAHL, M., KNIP, M., SAARMA, M. & LINDHOLM, P. 2016. Increased circulating concentrations of mesencephalic astrocyte-derived neurotrophic factor in children with type 1 diabetes. *Sci Rep,* 6**,** 29058.

GIRONA, J., RODRÍGUEZ-BORJABAD, C., IBARRETXE, D., VALLVÉ, J. C., FERRÉ, R., HERAS, M., RODRÍGUEZ-CALVO, R., GUAITA-ESTERUELAS, S., MARTÍNEZ-MICAELO, N., PLANA, N. & MASANA, L. 2019. The Circulating GRP78/BiP Is a Marker of Metabolic Diseases and Atherosclerosis: Bringing Endoplasmic Reticulum Stress into the Clinical Scenario. *J Clin Med,* 8.

GIUSTI, L., BALDINI, C., CIREGIA, F., GIANNACCINI, G., GIACOMELLI, C., DE FEO, F., DELLE SEDIE, A., RIENTE, L., LUCACCHINI, A., BAZZICHI, L. & BOMBARDIERI, S. 2010. Is GRP78/BiP a potential salivary biomarker in patients with rheumatoid arthritis? *Proteomics Clin Appl,* 4**,** 315-24.

KAKUGAWA, T., YOKOTA, S.-I., ISHIMATSU, Y., HAYASHI, T., NAKASHIMA, S., HARA, S., SAKAMOTO, N., KUBOTA, H., MINE, M., MATSUOKA, Y., MUKAE, H., NAGATA, K. & KOHNO, S. 2014. Serum heat shock protein 47 levels are elevated in acute interstitial pneumonia. *BMC pulmonary medicine,* 14**,** 48-48.

KANI, K., MALIHI, P. D., JIANG, Y., WANG, H., WANG, Y., RUDERMAN, D. L., AGUS, D. B., MALLICK, P. & GROSS, M. E. 2013. Anterior gradient 2 (AGR2): blood-based biomarker elevated in metastatic prostate cancer associated with the neuroendocrine phenotype. *Prostate,* 73**,** 306-15.

KIM, Y., LEE, H., MANSON, S. R., LINDAHL, M., EVANS, B., MINER, J. H., URANO, F. & CHEN, Y. M. 2016. Mesencephalic Astrocyte-Derived Neurotrophic Factor as a Urine Biomarker for Endoplasmic Reticulum Stress-Related Kidney Diseases. *J Am Soc Nephrol,* 27**,** 2974-2982.

KORF-KLINGEBIEL, M., REBOLL, M. R., KLEDE, S., BROD, T., PICH, A., POLTEN, F., NAPP, L. C., BAUERSACHS, J., GANSER, A., BRINKMANN, E., REIMANN, I., KEMPF, T., NIESSEN, H. W., MIZRAHI, J., SCHONFELD, H. J., IGLESIAS, A., BOBADILLA, M., WANG, Y. & WOLLERT, K. C. 2015. Myeloid-derived growth factor (C19orf10) mediates cardiac repair following myocardial infarction. *Nat Med,* 21**,** 140-9.

LIU, R., GONG, J., CHEN, J., LI, Q., SONG, C., ZHANG, J., LI, Y., LIU, Z., DONG, Y., CHEN, L. & JIN, B. 2012. Calreticulin as a potential diagnostic biomarker for lung cancer. *Cancer Immunology, Immunotherapy,* 61**,** 855-864.

MA, J., LUO, X., WU, Q., CHEN, Z., KOU, L. & WANG, H. 2010. Circulation levels of acute phase proteins in patients with Takayasu arteritis. *J Vasc Surg,* 51**,** 700-6.

MAKAWITA, S., SMITH, C., BATRUCH, I., ZHENG, Y., RÜCKERT, F., GRÜTZMANN, R., PILARSKY, C., GALLINGER, S. & DIAMANDIS, E. P. 2011. Integrated proteomic profiling of cell line conditioned media and pancreatic juice for the identification of pancreatic cancer biomarkers. *Molecular & cellular proteomics : MCP,* 10**,** M111.008599-M111.008599.

MERALI, S., BARRERO, C. A., BOWLER, R. P., CHEN, D. E., CRINER, G., BRAVERMAN, A., LITWIN, S., YEUNG, A. & KELSEN, S. G. 2014. Analysis of the plasma proteome in COPD: Novel low abundance proteins reflect the severity of lung remodeling. *COPD,* 11.

NGUYEN, E. V., GHARIB, S. A., PALAZZO, S. J., CHOW, Y.-H., GOODLETT, D. R. & SCHNAPP, L. M. 2013. Proteomic Profiling of Bronchoalveolar Lavage Fluid in Critically Ill Patients with Ventilator-Associated Pneumonia. *PLOS ONE,* 8**,** e58782.

POLTEN, F., REBOLL, M., WIDERA, C., KEMPF, T., BETHMANN, K., GUPTA, P., MIGLIETTA, J., PEKCEC, A., TILLMANNS, J., BAUERSACHS, J., GIANNITSIS, E., PICH, A. & WOLLERT, K. C. 2018. Plasma Concentrations of Myeloid-Derived Growth Factor in Healthy Individuals and Patients with Acute Myocardial Infarction as Assessed by Multiple Reaction Monitoring-Mass Spectrometry. *Anal Chem*.

RODRÍGUEZ-GONZÁLEZ, R., MILLÁN, M., SOBRINO, T., MIRANDA, E., BREA, D., DE LA OSSA, N. P., BLANCO, M., PEREZ, J., DORADO, L., CASTELLANOS, M., LOMAS, D. A., MORO, M. A., DÁVALOS, A. & CASTILLO, J. 2011. The natural tissue plasminogen activator inhibitor neuroserpin and acute ischaemic stroke outcome. *Thromb Haemost,* 105**,** 421-9.

SOGAWA, K., TAKANO, S., IIDA, F., SATOH, M., TSUCHIDA, S., KAWASHIMA, Y., YOSHITOMI, H., SANDA, A., KODERA, Y., TAKIZAWA, H., MIKATA, R., OHTSUKA, M., SHIMIZU, H., MIYAZAKI, M., YOKOSUKA, O. & NOMURA, F. 2016. Identification of a novel serum biomarker for pancreatic cancer, C4b-binding protein α-chain (C4BPA) by quantitative proteomic analysis using tandem mass tags. *British journal of cancer,* 115**,** 949-956.

SOLLAZZO, D., FORTE, D., POLVERELLI, N., PERRICONE, M., ROMANO, M., LUATTI, S., VIANELLI, N., CAVO, M., PALANDRI, F. & CATANI, L. 2016. Circulating Calreticulin Is Increased in Myelofibrosis: Correlation with Interleukin-6 Plasma Levels, Bone Marrow Fibrosis, and Splenomegaly. *Mediators of inflammation,* 2016**,** 5860657-5860657.

TAKAHASHI, K., PAVLIDIS, S., NG KEE KWONG, F., HODA, U., ROSSIOS, C., SUN, K., LOZA, M., BARIBAUD, F., CHANEZ, P., FOWLER, S. J., HORVATH, I., MONTUSCHI, P., SINGER, F., MUSIAL, J., DAHLEN, B., DAHLEN, S. E., KRUG, N., SANDSTROM, T., SHAW, D. E., LUTTER, R., BAKKE, P., FLEMING, L. J., HOWARTH, P. H., CARUSO, M., SOUSA, A. R., CORFIELD, J., AUFFRAY, C., DE MEULDER, B., LEFAUDEUX, D., DJUKANOVIC, R., STERK, P. J., GUO, Y., ADCOCK, I. M. & CHUNG, K. F. 2018. Sputum proteomics and airway cell transcripts of current and ex-smokers with severe asthma in U-BIOPRED: an exploratory analysis. *Eur Respir J,* 51.

TARR, J. M., WINYARD, P. G., RYAN, B., HARRIES, L. W., HAIGH, R., VINER, N. & EGGLETON, P. 2010. Extracellular calreticulin is present in the joints of patients with rheumatoid arthritis and inhibits FasL (CD95L)-mediated apoptosis of T cells. *Arthritis Rheum,* 62**,** 2919-29.

TRYCHTA, K. A., BACK, S., HENDERSON, M. J. & HARVEY, B. K. 2018. KDEL Receptors Are Differentially Regulated to Maintain the ER Proteome under Calcium Deficiency. *Cell Rep,* 25**,** 1829-1840.e6.

WANG, Y., XIE, J., LIU, Z., FU, H., HUO, Q., GU, Y. & LIU, Y. 2017. Association of calreticulin expression with disease activity and organ damage in systemic lupus erythematosus patients. *Experimental and therapeutic medicine,* 13**,** 2577-2583.

WU, T., ZHANG, F., YANG, Q., ZHANG, Y., LIU, Q., JIANG, W., CAO, H., LI, D., XIE, S., TONG, N. & HE, J. 2017. Circulating mesencephalic astrocyte-derived neurotrophic factor is increased in newly diagnosed prediabetic and diabetic patients, and is associated with insulin resistance. *Endocr J,* 64**,** 403-410.

ZUBAIR, M. & AHMAD, J. 2015. Plasma Heat Shock Proteins (HSPs) 70 and 47 levels in diabetic foot and its possible correlation with clinical variables in a North Indian Tertiary care hospital. *Diabetes Metab Syndr,* 9**,** 237-43.
