# Supplementary material for: Computational Modeling of C-Terminal Tails to Predict the Calcium-Dependent Secretion of Endoplasmic Reticulum Resident Proteins
Source: Front Chem. 2021 Jun 29;9:689608. doi: 10.3389/fchem.2021.689608 (PMC8276033; doi:10.3389/fchem.2021.689608)
Supplement: Supplementary file 6 [file Table2.DOCX]

**Supplementary Table S2: Pulmonary surfactant-associated protein B (SFTPB) and Contactin-2 (CNTN2) are increased extracellularly in disease states.**

| Protein | Model | C-terminus | Measurement | Ref |
| --- | --- | --- | --- | --- |
| Cancer | | | | |
| SFTPB | Tumor bearing mice from lung adenocarcinoma models | CFQTPHL | MS of plasma | (Taguchi et al., 2011) |
| SFTPB | Patients with diagnosed non-small cell lung carcinoma | CIHSPDL | ELISA of plasma | (Taguchi et al., 2011) |
| Lung Disorders | | | | |
| SFTPB | Patients diagnosed with acute respiratory distress syndrome | CIHSPDL | ELISA of plasma | (Doyle et al., 1997) |
| SFTPB | Patients diagnosed with acute cardiogenic pulmonary edema | CIHSPDL | ELISA of plasma | (Doyle et al., 1997) |
| SFTPB | Current smokers | CIHSPDL | ELISA of plasma | (Nguyen et al., 2011) |
| SFTPB | Infants with respiratory syncytial virus | CIHSPDL | ELISA of plasma | (Wang et al., 1999) |
| SFTPB | Current smokers | CIHSPDL | ELISA of serum | (Robin et al., 2002) |
| SFTPB | People before and after attendance at a chlorinated pool with nitrogen trichloride gas exposure | CIHSPDL | ELISA of serum | (Carbonnelle et al., 2002) |
| Ischemia |  |  |  |  |
| SFTPB | Patients with a cerebral infarction | CIHSPDL | ELISA of cerebrospinal fluid | (Schob et al., 2013) |
| Alzheimer’s disease (AD) | | | | |
| CNTN2 | AD patients | LIGSLEL | LC-MS/MS of cerebrospinal fluid | (Yin et al., 2009) |
| Heart disease | | | | |
| SFTPB | Patients with chronic heart failure | CIHSPDL | ELISA of plasma | (De Pasquale Carmine et al., 2004) |
| SFTPB | Patients with exercise-induced myocardial ischemia pre- and post-exercise | CIHSPDL | ELISA of plasma | (De Pasquale et al., 2005) |

MS, mass spectrometry; ELISA, enzyme linked immunosorbent assay; LC-MS/MS, liquid chromatography tandem mass spectrometry

**References**

CARBONNELLE, S., FRANCAUX, M., DOYLE, I., DUMONT, X., DE BURBURE, C., MOREL, G., MICHEL, O. & BERNARD, A. 2002. Changes in serum pneumoproteins caused by short-term exposures to nitrogen trichloride in indoor chlorinated swimming pools. *Biomarkers,* 7**,** 464-78.

DE PASQUALE CARMINE, G., ARNOLDA LEONARD, F., DOYLE IAN, R., AYLWARD PHILIP, E., CHEW DEREK, P. & BERSTEN ANDREW, D. 2004. Plasma Surfactant Protein-B. *Circulation,* 110**,** 1091-1096.

DE PASQUALE, C. G., ARNOLDA, L. F., DOYLE, I. R., AYLWARD, P. E., RUSSELL, A. E. & BERSTEN, A. D. 2005. Circulating surfactant protein-B levels increase acutely in response to exercise-induced left ventricular dysfunction. *Clin Exp Pharmacol Physiol,* 32**,** 622-7.

DOYLE, I. R., BERSTEN, A. D. & NICHOLAS, T. E. 1997. Surfactant proteins-A and -B are elevated in plasma of patients with acute respiratory failure. *Am J Respir Crit Care Med,* 156**,** 1217-29.

NGUYEN, A. B., ROHATGI, A., GARCIA, C. K., AYERS, C. R., DAS, S. R., LAKOSKI, S. G., BERRY, J. D., KHERA, A., MCGUIRE, D. K. & DE LEMOS, J. A. 2011. Interactions between smoking, pulmonary surfactant protein B, and atherosclerosis in the general population: the Dallas Heart Study. *Arteriosclerosis, thrombosis, and vascular biology,* 31**,** 2136-2143.

ROBIN, M., DONG, P., HERMANS, C., BERNARD, A., BERSTEN, A. D. & DOYLE, I. R. 2002. Serum levels of CC16, SP-A and SP-B reflect tobacco-smoke exposure in asymptomatic subjects. *Eur Respir J,* 20**,** 1152-61.

SCHOB, S., SCHICHT, M., SEL, S., STILLER, D., KEKULÉ, A., PAULSEN, F., MARONDE, E. & BRÄUER, L. 2013. The Detection of Surfactant Proteins A, B, C and D in the Human Brain and Their Regulation in Cerebral Infarction, Autoimmune Conditions and Infections of the CNS. *PLOS ONE,* 8**,** e74412.

TAGUCHI, A., POLITI, K., PITTERI, S. J., LOCKWOOD, W. W., FAÇA, V. M., KELLY-SPRATT, K., WONG, C.-H., ZHANG, Q., CHIN, A., PARK, K.-S., GOODMAN, G., GAZDAR, A. F., SAGE, J., DINULESCU, D. M., KUCHERLAPATI, R., DEPINHO, R. A., KEMP, C. J., VARMUS, H. E. & HANASH, S. M. 2011. Lung cancer signatures in plasma based on proteome profiling of mouse tumor models. *Cancer cell,* 20**,** 289-299.

WANG, S. Z., DOYLE, I. R., NICHOLAS, T. E. & FORSYTH, K. D. 1999. Plasma surfactant protein-B is elevated in infants with respiratory syncytial virus-induced bronchiolitis. *Pediatric research,* 46**,** 731-734.

YIN, G. N., LEE, H. W., CHO, J.-Y. & SUK, K. 2009. Neuronal pentraxin receptor in cerebrospinal fluid as a potential biomarker for neurodegenerative diseases. *Brain Research,* 1265**,** 158-170.
